# Positive Classification Advantage: Tracing the Time Course Based on Brain Oscillation

**DOI:** 10.3389/fnhum.2017.00659

**Published:** 2018-01-11

**Authors:** Tianyi Yan, Xiaonan Dong, Nan Mu, Tiantian Liu, Duanduan Chen, Li Deng, Changming Wang, Lun Zhao

**Affiliations:** ^1^School of Life Science, Beijing Institute of Technology, Beijing, China; ^2^Beijing Key Laboratory of Network System Architecture and Convergence, Beijing University of Posts and Telecommunications, Beijing, China; ^3^Beijing Key Laboratory of Mental Disorders, Beijing Anding Hospital, Capital Medical University, Beijing, China; ^4^Institute of Brain Research, Beijing Yiran Sunny Technology Co., Ltd., Beijing, China

**Keywords:** positive classification, reaction times, brain oscillation, correlation, time intervals

## Abstract

The present study aimed to explore the modulation of frequency bands (alpha, beta, theta) underlying the positive facial expressions classification advantage within different post-stimulus time intervals (100–200 ms, 200–300 ms, 300–400 ms). For this purpose, we recorded electroencephalogram (EEG) activity during an emotion discrimination task for happy, sad and neutral faces. The correlation between the non-phase-locked power of frequency bands and reaction times (RTs) was assessed. The results revealed that beta played a major role in positive classification advantage (PCA) within the 100–200 and 300–400 ms intervals, whereas theta was important within the 200–300 ms interval. We propose that the beta band modulated the neutral and emotional face classification process, and that the theta band modulated for happy and sad face classification.

## Introduction

Facial expressions play an important role in social life. The information is valuable for interpreting how others feel and their behavioral tendencies. Ekman (1994) classified emotional facial expressions to six basic categories (happiness, sadness, anger, disgust, fear and surprise). So far, methods such as single cell recordings, functional brain imaging and event-related potentials (ERPs) have been used to investigate brain activity involving perception, emotion, behavior, etc. Thus, studies have shown the probable neural network of emotionally salient stimuli (Eimer and Holmes, 2007). As shown by previous studies, brain activities related to emotional events, including those in the higher order sensory cortex, amygdala, orbitofrontal cortex and ventral striatum, share complex interconnected structural network. However, much more research needed to understand the brain mechanisms underlying emotion.

The recognition speed of facial expressions of emotion is very easy to obtain. Abundant data from research has revealed that the recognition speed of happy faces is faster than sad faces (Crews and Harrison, 1994; Leppänen and Hietanen, 2004; Calvo and Beltrán, 2013; Liu et al., 2013), angry faces (Billings et al., 1993; Hugdahl et al., 1993; Calvo and Beltrán, 2013), disgusted faces (Stalans and Wedding, 1985) and neutral faces (Hugdahl et al., 1993; Leppänen and Hietanen, 2004; Calvo and Beltrán, 2013; Liu et al., 2013). However, behavioral experiments can only use simple measures such as performance accuracy and reaction times (RTs). In addition, the RTs of multiple expressions classification tasks are usually 1 s or above (e.g., Calder et al., 2000; Palermo and Coltheart, 2004; Calvo and Lundqvist, 2008), which, in terms of advance chronometry, are very large time scales. For exploring more precise time processes, previous studies have explored the neural mechanism of facial expression classification using ERPs (e.g., Eimer and Holmes, 2007; Lynn and Salisbury, 2008). Some ERP research has revealed the phenomenon of positive classification advantage (PCA), which means that positive facial emotional expressions are recognized faster than negative ones (Leppänen and Hietanen, 2004). PCA is strongly linked to late perceptual processing, the differences between fearful and happy faces were shown over occipital regions as early as 80 ms post-stimuli, and those between happy and sad faces between 90 ms and 110 ms. Thus, our research focuses on this time course of PCA processing.

Many previous studies have revealed that ERP components are strongly linked to the categorization of facial emotion expression. Studies have shown that PCA is related to late components (Liu et al., 2013). However, information on the time course of facial emotion categorization has not been revealed. Brain oscillations, which could better track the activities of neurons, could be an efficient way to explore the time course of facial emotional categorizations. Oscillation activities could provide key physiologically information of brain dynamics. Furthermore, we can use neurofeedback to train different oscillation activities. Thus, we can discriminate between positive stimuli more quickly. Thus, the electroencephalogram (EEG) dynamics of face perception and facial expression have recently been analyzed through oscillation dynamics.

Event-related theta oscillations (4–7 Hz) have been reported to play an important role in cognitive processes such as memory, attention and cognition (Klimesch et al., 1997; Kahana et al., 2001; Khader et al., 2010; Sauseng et al., 2010). Balconi and Lucchiari (2006) reported enhanced frontal theta synchronization to emotional facial expressions as compared, with neutral expressions. Additionally, higher theta synchronization to fearful facial expressions than neutral expressions was observed. Similarly, Knyazev et al. (2009) reported that theta synchronization was higher in response to emotional faces (angry and happy faces) than neutral faces (Knyazev et al., 2009).

Alpha oscillations (8–15 Hz), which are pronounced due to their asymmetric effect, have been studied for many years (Davidson, 2003, 2004; Coan and Allen, 2004; Herrmann et al., 2004). Despite these obvious features on emotional processing, Güntekin and Basar (2007) found that in comparison with happy expressions, angry expressions elicited higher alpha responses at T5, P3 and O2 electrodes. Additionally, in an MEG experiment by Onoda et al., they found that event-related alpha power in the occipital region is higher in negative conditions than in other conditions (neutral and positive; Onoda et al., 2007). However, Balconi and Mazza (2009) reported that compared with neutral stimuli, positive and negative emotions trigger decreased alpha power responses. Furthermore, they also found that alpha oscillation was associated with an increase in left hemisphere activity (Balconi and Mazza, 2010). Thus, the modulation of alpha oscillations on emotional processes is still not clear.

Beta oscillations (16–30 Hz) have been thought to have a strong link with sensorimotor functions and could be reduced by voluntary movements and motor imagery (Neuper et al., 2009; Engel and Fries, 2010). Some researchers have reported enhanced beta activities in response to affective stimuli compared with neutral stimuli (Woodruff et al., 2011). Güntekin and Başar (2010) also reported higher beta activity in response to negative images than positive images in frontal, central and parietal electrodes upon presentation of IAPS images (Güntekin and Başar, 2010). In addition, Schutter et al. (2001) conducted a spontaneous EEG study and found a significant relationship in response to angry facial stimuli between asymmetry in parietal beta power and the attentional response (Schutter et al., 2001). Güntekin and Basar (2007) also reported increased beta power in response to angry facial stimuli compared with happy stimuli at F3 and Cz (Güntekin and Basar, 2007). However, Zhang et al. (2013) found greater beta oscillation activity for positive facial expressions than for negative expressions. Emotion processing mechanisms are rather complicated to reveal; thus, more studies are needed to complete our knowledge on these related brain structures.

As mentioned above, the results on the brain oscillations produced upon the processing of emotional faces have been controversial. Thus, in the present study, we will trace the time course of PCA based on brain oscillations. Our goal is to find the modulation of PCA on frequency bands within different time intervals. In prior research, Liu et al. (2013) found the N170 (150–170 ms) component, posterior N2 (250–290 ms) component and P3 (350–450 ms) component using schematic face stimuli (Liu et al., 2013). Moreover, they found that neutral faces elicited a shorter N170 latency compared with happy and sad faces. Meanwhile, they found that happy faces elicited more negative N2 activity compared with neutral and sad faces. Additionally, happy and neutral faces elicited higher P3 amplitudes and shorter P3 latencies compared with sad faces. Based on these results, we assumed that when a subject makes a decision, he/she first classifies the neutral face (time window 1: 100–200 ms) and then discriminates between happy and sad expressions (time window 2: 200–300 ms and time window 3: 100–200 ms). Thus, the time course of PCA is time window 2 and 3. We will mainly focus on these two time windows.

To test our hypothesis, we used schematic face stimuli based on Liu et al. (2013). Schematic faces allow us to control physical features as carefully as possible, to minimize influence from additional information related to facial identity (e.g., gender, race) and to exclude the confounding effects of general arousal as well as valence, *per se* (Boucsein et al., 2001; Eger et al., 2003; Krombholz et al., 2007; Babiloni et al., 2010). Moreover, Sagiv and Bentin (2001) have proved that even schematic faces (only made from simple line fragments) could trigger face-sensitive N170, and that this effect was not attributable to an artifact arising from facilitated recognition of a single feature (Sagiv and Bentin, 2001; Leppänen and Hietanen, 2004). In addition, schematic face stimuli are reported to be able to provide emotional stimuli; significant increase of fMRI signal can be found in the amygdala, hippocampus and prefrontal cortex in response to emotional vs. neutral schematic faces (Wright et al., 2002), indicating the feasibility for applying the schematic faces to study PCA.

## Materials and Methods

### Subjects

This study was carried out in accordance with the recommendations of “School of Life Science Ethics Committee, Beijing Institute of Technology” with written informed consent from all subjects. All subjects gave written informed consent in accordance with the Declaration of Helsinki. The protocol was approved by the “School of Life Science Ethics Committee, Beijing Institute of Technology”. Eighteen young healthy individuals participated in our study (10 females; 20–25 years of age; mean: 22.6 years). All participants were right-handed, had normal or corrected-to-normal visual acuity and were free of a neurological or psychiatric history. They received payments for their participation and gave their written informed consent before the experiment (Liu et al., 2013); however, the study only examined PCA in the classic time-amplitude domain and no time-frequency analyses have been previously reported.

### Stimuli and Procedure

**Figure 1:**
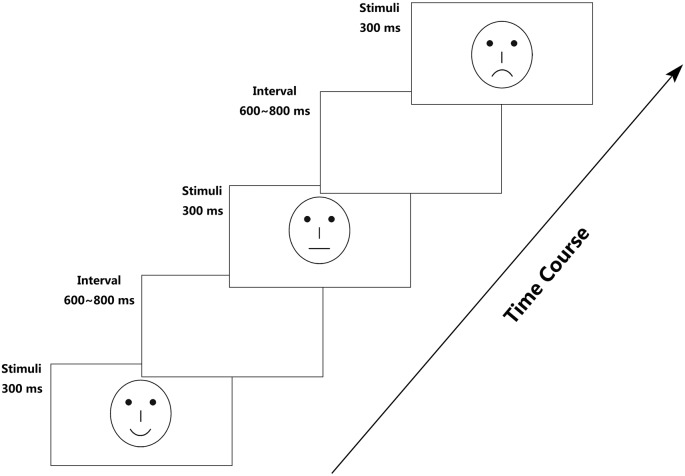
Example stimuli of schematic facial expressions.

To avoid the low-level processing of facial features, as well as boredom by the excessive repetition of one single model, each facial expression category consisted of 18 different schematic face models by manipulating the distance among facial features and by manipulating the shape of the facial features, particularly the mouths. Figure 1 illustrates examples of schematic face expressions we used as stimuli. All stimuli were presented at the center of a cathode ray tube video monitor and were viewed from a distance of 100 cm at a visual angle of approximately 7.27° × 6.06°.

Following electrode application, the participants were seated in a dimly lit and sound-attenuated cabin. They were instructed to classify each face by the expression it represented (happy, neutral, or sad) and to respond by pressing correspondingly labeled buttons on the keyboard with the left index finger (Z key), right index finger (N key), or right middle finger (M key). Speed and accuracy were equally emphasized (Liu et al., 2013). All of the 324 stimuli (3 facial expressions × 108 faces) were randomly presented in a mixed design, with three blocks, each of which possess 108 stimuli and a short break in between. To offset the difference between the fingers, the labels of the response buttons (happy–neutral–sad/sad–happy–neutral/neutral–sad–happy) counterbalanced across the participants. Each face was presented for 300 ms with an inter-trial interval ranging randomly between 600 ms and 800 ms, starting after response.

The participants completed one practice sequence of 18 stimuli (six from each type, equally representing the three facial expressions). These stimuli were not used in the main experiment, which lasted approximately 15 min.

### EEG Recording

An EEG was recorded continuously using an electrode cap with 64 sintered Ag/AgCI electrodes mounted according to the extended international 10–20 system and referenced to the tip of the nose. An electrooculogram (EOG) was recorded via two pairs of additional electrodes, with one placed above and below the left eye and the other placed to the external canthi of both eyes. The EEG and EOG were amplified and digitized by the NeuroLab Amplifier (Yiran Sunny Technology Co. Ltd., Beijing, China) with a bandpass of 0.05–100 Hz and a sampling rate of 500 Hz. Electrode impedance was kept below 5 kΩ throughout the experiment.

### Data Analysis

Data analysis was performed using MATLAB R2013a (Mathworks Inc., Natick, MA, USA) with the open source toolboxes EEGLAB (Swartz Center for Computational Neuroscience, La Jolla, CA, USA)^1^. The artifacts (e.g., eye artifacts, muscle artifacts and electrocardiographic activity) of all channels were removed by independent component analysis (ICA). After the artifact correction of EEF data, epochs (600 ms pre- to 900 ms post-stimulus onset) were sorted according to stimulus condition to create a plot of time-frequency representations (TFRs). Total frequency band responses were analyzed via a Morlet wavelet using the MATLAB wavelet toolbox (MathWorks). Morlet c was set to 7, and the final power was μV^2^. The TFRs of the theta band power of each participant were calculated; these ranged from 4 Hz to 7 Hz, whereas the alpha band ranged from 8 Hz to 15 Hz, and the beta band from 16 Hz to 30 Hz. We calculated the synchrony among the medial, right, and left electrodes and subtracted the frequency-specific baseline (−300 to 0 ms pre-stimulus). Wavelet activity was individually returned by wavelet decomposition for each trail. Changes in the amplitude of activity were measured every 100 ms from 100 ms to 400 ms post-stimuli (e.g., 100–200 ms post-stimuli, 200–300 ms post-stimuli, 300–400 ms post-stimuli …) to cover a whole cycle of the high frequencies.

Accuracy rates and RTs (from the stimulus onset) were recorded and analyzed using a one-way ANOVA design, with expression (happy, neutral and sad) as the within- subjects factor. Based on previous studies, for each EEG frequency band the measurements were analyzed using a repeated-measures ANOVA treating facial expressions (happy, neutral and sad), hemisphere (left, right), and site (P7/8, PO7/8, P9/10) as within-subject factors. For factors with more than two levels, the degrees of freedom were corrected using the Greenhouse-Geisser procedure (for simplicity, the uncorrected degrees of freedom are presented). *Post hoc* comparisons were performed with the Bonferroni procedure.

## Results

### Performance

A one-way ANOVA analysis was conducted for the percentage of correct responses. The main effect of expression was significant, *F*_(2,34)_ = 7.95, *p* = 0.003, partial *η*^2^ = 0.319. *Post hoc* comparisons showed that neutral faces were identified more correctly (97.4%) than either happy faces (93.7%, *p* = 0.002) or sad faces (94.2%, *p* = 0.007), with no differences between the latter conditions (*p* > 0.9). For each participant, incorrect responses or responses with RTs more than ±2 SDs from the mean in each condition were excluded for RT analysis. On average, 8.7% of the responses were removed. The RTs were analyzed by using the same statistical model as that for percentages of correct responses. There was a significant main effect of expression, *F*_(2,34)_ = 95.2, *p* < 0.001, partial *η*^2^ = 0.849, showing that neutral face categorization was faster (551 ms) than happy face categorization (602 ms, *p* < 0.001), which was quicker than classifying sad faces (656 ms, *p* < 0.001). To investigate the possible source of the PCA, a Pearson correlation analysis was conducted. This comparison showed that there was an overall significant positive correlation between the RT to negative face stimuli and the size of the PCA, *r* = 0.66, *p* < 0.005 (two tailed), but not between the RTs to positive face stimuli and the PCA, *r* = 0.17, *p* > 0.05.

### Time-Frequency Analysis

**Figure 2:**
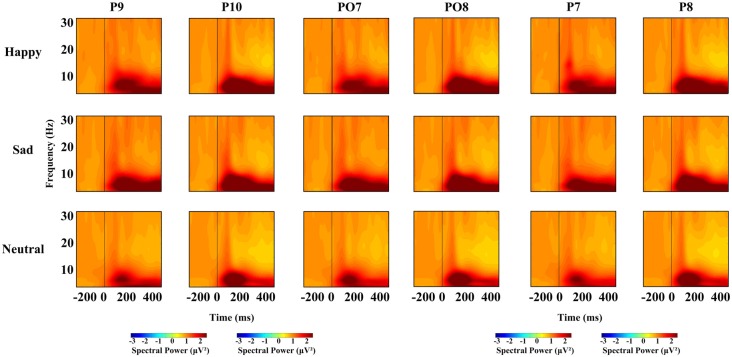
Spectral power for happy, sad and neutral expressions on different sites (P9, P10, PO7, PO8, P7 and P8).

For each time window and each frequency, a repeated-measures ANOVA with facial expression (happy, neutral and sad), and hemisphere (left, right) was used to examine the overall effects for different oscillation (theta, alpha, beta), respectively. We conducted four ANOVAs, one for each time interval. Based on previous research, we conducted a repeated-measures ANOVA with within-subject factors as facial expression (happy, neutral and sad), hemisphere (left, right) and site (P7/8, PO7/8 and P9/10), and different frequency bands, were used at lateral posterior sites (left, P7, PO7 and P9; right, P8, PO8 and P10). For all the ANOVAs, the degrees of freedom were Greenhouse-Geisser corrected where appropriate. Figure 2 shows the spectral power for happy, sad and neutral expressions on P9, P10, PO7, PO8, P7, P8, respectively.

### Oscillation Activities

**Figure 3:**
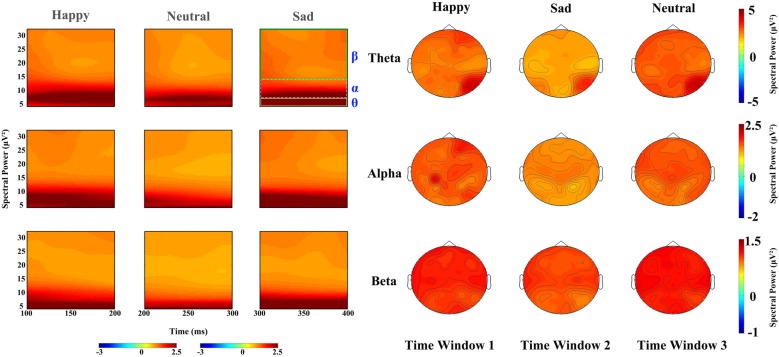
Left: spectral power for happy, sad and neutral expressions on time window 1 (100–20 ms post-stimuli), time window 2 (200–300 ms post-stimuli) and time window 3 (300–400 ms post-stimuli), respectively; Right: power topography for happy, sad and neutral expressions on theta, alpha and beta band and time window 1 (100–200 ms post-stimuli), time window 2 (200–300 ms post-stimuli) and time window 3 (300–400 ms post–stimuli), respectively.

In the 100–200 ms time window, the main effects for expressions on theta band revealed that sad faces (2.757 μV^2^) elicited lower power than happy (3.126 μV^2^, *p* < 0.01) and neutral faces (3.148 μV^2^, *p* < 0.02), with no difference between latter two conditions, and that the power elicited by sad expressions was lower than happy (*p* < 0.01) and neutral expressions (*p* < 0.001) on alpha band (*F*_(2,34)_ = 11.294, *p* < 0.001, partial *η*^2^ = 0.399) and beta band (*F*_(2,34)_ = 14.699, *p* < 0.001, partial *η*^2^ = 0.464) with no difference between the latter two conditions. The main effect of hemisphere was also significant on theta (*F*_(1,17)_ = 10.643, *p* < 0.01, partial *η*^2^ = 0.385) and beta band (*F*_(1,17)_ = 8.056, *p* < 0.02, partial *η*^2^ = 0.322), revealing a right hemisphere dominance (3.634 μV^2^ and 2.587 μV^2^ for left and right hemisphere, respectively) on the theta band, while power at left occipital sites (1.029 μV^2^) were larger than at right occipital sites (0.97 μV^2^) on beta band. Figure 3 shows the power for happy, sad and neutral expressions on difference time windows and different frequencies. There was no significant two-way interaction between factors (expression, hemisphere, site) on difference time windows and different frequencies (please refer to Supplementary Table S1).

For the time window from 200 ms to 300 ms, theta band showed a significant difference in facial expressions (*F*_(2,34)_ = 37.96, *P* < 0.001, partial *η*^2^ = 0.691), revealing a higher power for happy faces (3.102 μV^2^) than neutral faces (2.86 μV^2^, *p* < 0.05) and sad faces (2.231 μV^2^, *p* < 0.01). The effect of hemisphere for theta band also showed a significant difference (*F*_(1,17)_ = 8.473, *P* < 0.01, partial *η*^2^ = 0.322), with the hemisphere being more prominent (2.965 μV^2^) than the left (2.497 μV^2^). Alpha oscillations also showed a significant difference in facial expressions (*F*_(2,34)_ = 9.681, *P* < 0.001, partial *η*^2^ = 0.363), revealing that sad faces elicited lower activity (1.171 μV^2^) than the happy (1.509 μV^2^, *p* < 0.01) and neutral faces (1.458 μV^2^, *p* < 0.01), but that there were no differences between the latter two types of faces (*p* = 0.948). There was a significant main effect of expression on beta band (*F*_(2,34)_ = 14.324, *P* < 0.001, partial *η*^2^ = 0.457), showing less sad activity (0.876 μV^2^) than neutral activity (0.999 μV^2^, *p* < 0.001) and happy activity (0.965 μV^2^, *p* < 0.01), and no difference between the latter two conditions. Hemisphere also showed a significant difference in beta band (*F*_(1,17)_ = 5.977, *P* < 0.05, partial *η*^2^ = 0.26), revealing a higher power at the left hemisphere (0.987 μV^2^) than that at the right (0.907 μV^2^).

The time window from 300 ms to 400 ms showed significant main differences in facial expression for theta (*F*_(2,34)_ = 49.263, *p* < 0.001, partial *η*^2^ = 0.743), alpha (*F*_(2,34)_ = 20.695, *p* < 0.001, partial *η*^2^ = 0.495), beta (*F*_(2,34)_ = 48.283, *p* < 0.001, partial *η*^2^ = 0.74), revealing that neutral and happy expressions elicited higher power than sad expressions (*p* < 0.001), while there was no difference between neutral and happy expressions. A significant main effect of hemisphere also found on alpha (*F*_(1,17)_ = 7.936, *p* < 0.02, partial *η*^2^ = 0.318) and beta band (*F*_(1,17)_ = 15.907, *p* < 0.001, partial *η*^2^ = 0.483), showing a left hemisphere dominance (1.204 μV^2^ and 1.064 μV^2^ for the left and right hemisphere on alpha band, 0.911 μV^2^ and 0.817 μV^2^ on beta band, respectively) on both alpha and beta bands.

### Pearson Correlations

**Figure 4:**
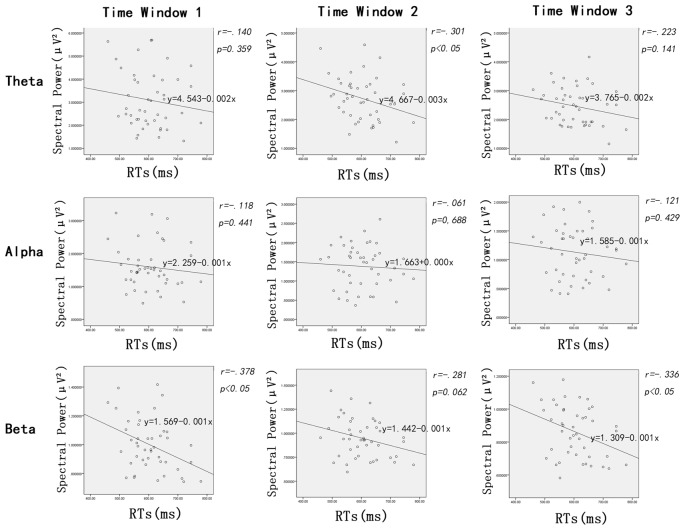
Scatter distribution of power of different frequencies and reaction time (RTs) during time window 1 (100–200 ms post-stimuli), time window 2 (200–300 ms post stimuli) and time window 3 (300–400 ms post-stimuli), respectively.

In addition to the ANOVAs, because we want to explore how the oscillation activities reflect modulations of the stimulus evaluation and decision processes, Pearson correlations between the RTs and power of all frequency bands (theta, alpha and beta, respectively) were conducted. Figure 4 shows scatterplot diagrams and linear fitting of the scatter-plots.

The Pearson correlations between RTs and the power of frequency bands showed significant negative correlations between RTs and beta power during the time window of 100–200 ms (*r* = −0.378, *p* < 0.02) and 300–400 ms (*r* = 0.336, *p* < 0.03); that is, the longer the RTs, the lower the beta power within time window 1 and 3. Interestingly, power on theta band was also negatively linked to RTs, but the time window was 200–300 ms (*r* = −0.301, *p* < 0.05). Furthermore, there were no significant correlations between the RTs and power of other oscillation bands (i.e., alpha) modulated by facial expressions (*p*s > 0.06).

## Discussion

Below is a summary of the results of the present research. Through facial expression classification experiments, we will discuss the phenomenon of happy face classification advantage using oscillation characteristics as well as time course. In line with previous studies, we found that face expression classification is quicker for happy expressions than for sad expressions (e.g., Crews and Harrison, 1994; Leppänen and Hietanen, 2004), and faster for neutral faces than happy faces. Moreover, happy faces elicited a higher spectral power than sad face. In accordance with other studies (Balconi and Mazza, 2009), the results showed a left hemisphere dominance, revealing the lateralization effect of positive expression classification. Although facial expressions elicited different activity on theta, alpha and beta frequencies, only beta and theta showed significant negative correlation with RT. Moreover, beta was strongly correlated with PCA in time window 1 and 3, whereas theta was correlated with time window 2. Additionally, the greater spectral power was linked to a shorter RT in all the three time windows and three frequency bands. Thus, quick responses require more brain activity, which was not obvious in the alpha bands.

In the present task, the theta oscillations showed that happy faces elicit higher power than sad faces, but the power elicited by neutral faces was higher than emotional faces (happy and sad faces). Previous studies with IAPS affective pictures as stimuli showed that emotional stimuli always elicited higher power than neutral stimuli (Balconi and Lucchiari, 2006; Zhang et al., 2013), which is in contrast with our results, but the stimuli in these experiments are not facial expressions. Studies using emotional video clips showed that power elicited by positive stimuli is higher than that by negative stimuli in the post-occipital area (Aftanas et al., 1998), which is similar to our results. Moreover, previous study using real emotional expressions suggested that happy expressions has faster results and higher accuracy than neutral expressions in discriminating tasks (Dasilva et al., 2016), which indicated the PCA.

As previously shown, alpha power showed an increase with positive stimuli in comparison with neutral stimuli after 100 ms post-stimuli, and although this difference is not significant, it is in line with previous studies that emotional stimuli elicits higher power than neutral stimuli. However, we found that in comparison with happy and neutral stimuli, power elicited by sad stimuli was the lowest. Interestingly, a study by Baumgartner et al. (2006) found that, when presenting IAPS pictures of fear, happiness and sadness, there were no differences in alpha power, but decreased alpha power was found when stimuli were emotional pictures accompanied with emotional music. For studies that found that negative stimuli elicits higher alpha responses, because negative stimuli in these studies were always angry pictures or affective pictures, the decreased alpha oscillations may be attributed to the facial classification mechanisms; however, whether real facial expressions would have same results as schematic facial expressions requires further study.

Previous studies on application of IAPS images found that negative images elicit greater beta responses compared with positive images in frontal, central and parietal electrodes (Güntekin and Başar, 2010); however, the stimuli they used were not facial expression pictures but affective pictures. A study by Zhang et al. (2013) with Chinese affective pictures as stimuli indicated that adolescents at the age of 12 exhibit more beta event-related synchronization (ERS) for positive vs. neutral stimuli. Other studies also verified that higher beta responses elicited both positive and negative stimuli than neutral stimuli (Miskovic and Schmidt, 2010; Cohen et al., 2013).

The ERP results have shown components (i.e., P1, N1, N170, P2, N2, P3) that have a strong relationship with facial emotion classification tasks. First, enhanced N170 (a negative ERP component during 140–180 ms post-stimuli at occipito-temporal electrodes), which is thought to be an indicator of inverted-face recognition, shows significant differences to fearful faces than neural faces at 160 ms post-stimuli (Holmes et al., 2005). Compared with time window 1, we concluded that face categorization was pre-attended during this time window. For posterior N2, a negativity peaking between 200 ms and 300 ms may be modulated by factors influencing visual stimuli categorization such as mutual information level, determined by gross similarity between the fragment and image in an image patch (Harel et al., 2007). In addition, as a generic name for relatively late positive component with a distribution at centro-parietal or centro-frontal midline area, P3 is considered in conjunction with facial emotion categorization (Polich, 2007). It has shown higher amplitudes and shorter latencies in response to both happy and neutral stimuli than sad stimuli, while RT displayed a significant correlation with amplitude and latency of the P3. During time windows 2 and 3, which are linked to the N2 and P3 components, emotion categorization is completed. Thus, the time-division can help us to better analyze the process of emotion categorization.

Though there were significant differences in theta, alpha and beta oscillations for different emotional stimuli (happy, neutral and sad), only theta and beta band significantly were correlated with RTs. However, the correlation between beta oscillation and RTs was as early as 100 ms after stimuli onset. That is, beta oscillation may modulate the categorization of neutral faces. In our results, the beta band had a major impact upon face discrimination. Also, the categorization occurred as early as 100 ms after stimulus onset, which has important effects for our social life. Furthermore, researchers have revealed that beta oscillation contributed to classification of known faces and unknown faces within 100–200 ms (Ozgören et al., 2005). Based on these results, we propose that in time window 1, subjects can discriminate between neutral and emotional faces, and that beta band is related to the process. This suggests that neutral face may be classified the fastest; and is modulated by beta band.

As for time windows 2 and 3, previous studies revealed that theta power was related to the P3 component (see a review Polich, 2007) and that P3 was related to PCA (Liu et al., 2013). That is, theta (time window 2) and beta (time window 3) oscillations modulate the PCA. These results are consistent with other research that focused on negative stimuli (Cohen et al., 2013). This research demonstrated that the theta band contributed to early emotion selection (200–300 ms) and that the beta band was a late response (400–600 ms). Thus, our hypothesis was verified, meaning that the theta band and beta band had a major impact on PCA.

In conclusion, our research used schematic emotional faces and focused on the time course of the recognition advantage of happy faces. The results demonstrate that the categorization process was mainly associated with beta oscillations from the beginning, while theta oscillations participated during the later period. Thus, we could detect PCA as early as 200 ms post-stimuli through theta oscillations, earlier than the other findings on time domain (usually 300 ms). Moreover, we can train theta bands in time window 2 and beta bands in time window 3, so that people can discriminate happy faces more quickly. This may have good implications for depressed patients. Therefore, advanced research on facial emotional processing is clearly needed.

## Author Contributions

TY contributed to the conception of the study. TL and XD performed the data analyses and wrote the manuscript. NM and DC contributed significantly to analysis and manuscript preparation. DC and LZ helped perform the analysis with constructive discussions. LD and CW provided data processing ideas and creative methods.

## Conflict of Interest Statement

The authors declare that the research was conducted in the absence of any commercial or financial relationships that could be construed as a potential conflict of interest.

## Footnotes

^1^http://sccn.ucsd.edu/eeglab/
