# Supplementary material for: Positive Classification Advantage: Tracing the Time Course Based on Brain Oscillation
Source: Front Hum Neurosci. 2018 Jan 11;11:659. doi: 10.3389/fnhum.2017.00659 (PMC5768652; doi:10.3389/fnhum.2017.00659)
Supplement: Supplementary file 1 [file Table_1.DOCX]

Supplementary Material

Positive classification advantage: Tracing the time course based on brain oscillation

Tianyi Yan1*, Tiantian Liu1, Nan Mu1 and Lun Zhao2,3*

*** Correspondence:** Tianyi Yan, Pro: [yantianyi@bit.edu.cn](mailto:yantianyi@bit.edu.cn); Lun Zhao, PhD: [zhaolunlun@aliyun.com](mailto:zhaolunlun@aliyun.com).

| Time window | Frequency | Expression*hemisphere | Expression*site | Hemisphere*site | Expression*hemisphere*site |
| --- | --- | --- | --- | --- | --- |
| 100-200ms | theta | F(2,34)=5.751  P=0.090 | F(4,68)=1.079  P=0.361 | F(2,34)=1.336  P=0.276 | **F(4,68)=4.465**  **P=0.024** |
|  | alpha | F(2,34)=1.713  P=0.199 | F(4,68)=0.337  P=0.635 | F(2,34)=2.736  P=0.104 | F(4,68)=2.985  P=0.080 |
|  | Beta | F(2,34)=0.310  P=0.725 | F(4,68)=1.250  P=0.300 | F(2,34)=3.821  P=0.069 | F(4,68)=1.833  P=0.183 |
| 200-300ms | theta | F(2,34)=3.158  P=0.066 | F(4,68)=1.695  P=0.182 | F(2,34)=0.250  P=0.703 | **F(4,68)=5.359**  **P=0.004** |
|  | alpha | F(2,34)=2.007  P=0.156 | F(4,68)=2.654  P=0.079 | F(2,34)=2.177  P=0.129 | **F(4,68)=4.770**  **P=0.006** |
|  | Beta | F(2,34)=0.988  P=0.381 | F(4,68)=0.767  P=0.473 | F(2,34)=1.572  P=0.227 | F(4,68)=1.082  P=0.362 |
| 300-400ms | theta | F(2,34)=1.213  P=0.303 | F(4,68)=2.329  P=0.103 | F(2,34)=1.215  P=0.308 | **F(4,68)=4.766**  **P=0.016** |
|  | alpha | F(2,34)=0.706  P=0.465 | F(4,68)=2.007  P=0.145 | F(2,34)=0.912  P=0.403 | F(4,68)=0.983  P=0.375 |
|  | Beta | F(2,34)=0.040  P=0.959 | F(4,68)=0.958  P=0.416 | F(2,34)=1.572  P=0.228 | F(4,68)=0.163  P=0.796 |

**Supplementary Table 1.** Interaction between factors (expression, hemisphere, site) within-subjects Effects

Bold values described significant differences (p<0.05) of interaction between factors (expression, hemisphere, site) within-subjects effects.
